# Apocynin Ameliorates Monosodium Glutamate Induced Testis Damage by Impaired Blood-Testis Barrier and Oxidative Stress Parameters

**DOI:** 10.3390/life13030822

**Published:** 2023-03-17

**Authors:** Merve Acikel-Elmas, Salva Asma Algilani, Begum Sahin, Ozlem Bingol Ozakpinar, Mert Gecim, Kutay Koroglu, Serap Arbak

**Affiliations:** 1 Department of Histology and Embryology, School of Medicine, Acibadem Mehmet Ali Aydinlar University, Icerenkoy Mah., Kayisdagi Cad. No. 32, Atasehir, Istanbul 34752, Turkey; 2 Department of Biochemistry, Faculty of Pharmacy, Marmara University, Basibuyuk Yolu, 4/A, Basibuyuk, Istanbul 34854, Turkey; 3 Department of Histology and Embryology, School of Medicine, Marmara University, Basibuyuk Yolu No. 9 D:2, Maltepe, Istanbul 34854, Turkey

**Keywords:** monosodium glutamate, apocynin, NOX-2, blood-testis barrier, ultrastructure

## Abstract

Background: the aim of this study was to investigate the effects of apocynin (APO) on hormone levels, the blood-testis barrier, and oxidative biomarkers in monosodium glutamate (MSG) induced testicular degeneration. Methods: Sprague Dawley male rats (150–200 g; n = 32) were randomly distributed into four groups: control, APO, MSG, and MSG + APO. MSG and MSG + APO groups were administered MSG (120 mg/kg) for 28 days. Moreover, the APO and MSG + APO groups received APO (25 mg/kg) during the last five days of the experiment. All administrations were via oral gavage. Finally, biochemical analyses were performed based on the determination of testosterone, follicle-stimulating hormone (FSH), luteinizing hormone (LH), malondialdehyde (MDA), glutathione (GSH), and superoxide dismutase (SOD), as well as light and transmission electron microscopic examinations, assessment of sperm parameters, ZO-1, occludin, NOX-2, and TUNEL immunohistochemistry were evaluated. Results: MSG increased both the oxidative stress level and apoptosis, decreased cell proliferation, and caused degeneration in testis morphology including in the blood-testis barrier. Administration of apocynin reversed all the deteriorated morphological and biochemical parameters in the MSG + APO group. Conclusions: apocynin is considered to prevent testicular degeneration by maintaining the integrity of the blood-testis barrier with balanced hormone and oxidant/antioxidant levels.

## 1. Introduction

The widely used L-glutamic acid, as an additive, occurs naturally in a variety of foods and is the source of the flavor enhancer, monosodium glutamate (MSG) [1]. Many processed foods contain MSG as an additive, with an average daily intake in European industrialized nations as 0.3 to 1.0 g. [2]. Even though MSG consumption is considered safe by food safety organizations, it is still questioned in several preclinical and clinical studies, especially its long-term exposure. Among the physiological functions of glutamate is its neurotransmitter property in the central nervous system, as a precursor of metabolites such as glutathione [1]. Long-term consumption of MSG has been shown to have negative effects on the male reproductive system. Studies have shown that long-term consumption of MSG decreases sperm count in males and affects the morphological structure of sperm and the testes [3]. The majority of the research cited previously focused primarily on the toxicity of MSG in rats at doses of 2000–8000 mg/kg body weight, which is extremely unlikely for humans at this level [4]. Allometric conversion by Shin et al. [5] showed this dose to be equivalent to 120 mg/kg body weight in rats. There are a significant number of glutamate receptors in the reproductive organs and sperm, making them susceptible to excitation damage from excess glutamate in the body. This makes the reproductive system a frequent target for glutamate-induced damage. In addition, glutamate toxicity is known to directly damage the hypothalamic–pituitary–gonadal axis, leading to a homeostatic imbalance in reproduction [6]. MSG has resulted in oxidative damage (increased lipid peroxidation and decreased antioxidant enzyme activities) and spermatogenic changes manifested by low sperm count and morphological abnormalities [3]. Therefore, we studied the effects of oral consumption of MSG on rats when they ingested a moderate dose extrapolated directly from the daily intake in humans. This MSG dose effect on rat testicular morphology was also observed in experimental studies [3,7]. The overproduction of reactive oxygen species (ROS) triggers oxidative stress and apoptosis. Due to their reactive structures, free radicals can interact with lipids, nucleic acids, and proteins and have harmful effects on the body [8]. Oxidative stress, induced by ROS, is known to play an important role in male infertility [9]. Studies have shown that NADPH oxidase (NOX) is one of the main sources of ROS [10]. Excessive ROS and oxidative stress in the male reproductive system cause negative changes in sperm concentration, motility, and morphology. Degenerated sperm and impaired semen parameters lead to infertility [11]. ROS causes male infertility due to DNA damage in the sperm [12]. Oxidative stress compromises the integrity of the plasma membrane of sperm and induces early capacitation. As a result, the fertilizing ability of sperm decreases and infertility occurs [13].

Apocynin (APO), extracted from the roots of the plant *Apocynum cannabinum*, is known to be effective as an inhibitor of NOX [14]. The anti-inflammatory effect of APO has been demonstrated in many experimental studies [15]. NOX activation occurs through the migration of cytosolic components to the cell membrane [16,17]. APO acts as a selective inhibitor of ROS production by acting on NOX activity in active human neutrophils. However, APO does not affect phagocytosis or other mechanisms of intracellular death [18,19]. NOX isoforms are expressed in various cells and have different physiological functions. NOX-2 is an isoform of NOX, which is present in eosinophils, macrophages, and neutrophils [20].

The spermatogenic cells are connected to each other and the Sertoli cells. This dynamic relationship is regulated by tight junctions and gap junctions [21]. The tight junctions between the Sertoli cells are important for the formation and function of the blood-testis barrier (BTB). The structure of the BTB includes tight junctions, desmosomes, basal ectoplasmic specializations, and gap junctions. Tight junctions of epithelial origin are multimolecular membrane specializations that contain multiple integral membrane proteins such as zonula occludens-1 (ZO-1) and occludin [21]. Occludins are one of the molecules contributing to the formation of tight junctions. Zonula occludens proteins such as zonula occludens-1 (ZO-1), ZO-2, and ZO-3 are important proteins involved in this structure. Occludin, ZO-1, and ZO-3 interact with the actin cytoskeleton. The protein ZO-3 is associated with the cytoplasmic domains of ZO-1 and occludin [22].

Oxidative stress occurs when there are not enough antioxidants against free radicals. A decrease in the activity of antioxidant enzymes such as superoxide dismutase (SOD) and glutathione (GSH) levels leads to an increase in oxidative damage [23]. Malondialdehyde (MDA), a parameter of cell membrane damage, indicates the density of oxygen radical attack in reactive cells and free radical metabolism in vivo. Decreased SOD and increased MDA trigger oxidative stress and cause cell damage, resulting in cell death [24]. Studies indicated NOX-2 as the source of ROS [20,25]. APO, a potent NOX-2 inhibitor [26], could be effective in the inhibition of NOX-2 to prevent tissue damage due to the oxidative stress. The administration of APO could have a curative effect on oxidative stress parameters and histopathological damage.

The aim of this study was to evaluate the effects of MSG on testicular tissue damage, cell proliferation, apoptosis, and oxidative stress indicated by the expression of NOX-2 and on the blood-testicular barrier. In addition, the study aimed to investigate whether apocynin can improve the effects on all these parameters.

## 2. Materials and Methods

### 2.1. Experimental Design

This experimental study was approved by the Ethics Committee of Acibadem Mehmet Ali Aydinlar University Experimental Animals (ACU-HADYEK, Approval number: HDK-2020/39). In this study, 8-week-old Sprague Dawley male albino rats (n = 32) were kept in cages with a temperature of 22 ± 2 °C and a standard light/dark (12:12 h) cycle. Rats were fed with standard animal food ad libitum for the 28 days of the experimental period. This research was conducted in accordance with the guidelines and regulations of ARRIVE (Animal Research: Reporting of In Vivo Experiments).

In this study, Sprague Dawley male rats (n = 8 in each group) were randomly divided into 4 groups as control, APO, MSG, and MSG + APO. Distilled water (1 mL) was given to the control group of rats by oral gavage for 28 days. The MSG and MSG + APO groups were administered 120 mg/kg MSG by oral gavage for 28 consecutive days [4]. The APO and the MSG + APO groups were administered 25 mg/kg APO by oral gavage on the last 5 days of the experiment [27]. The weights of the rats in the experimental groups were analyzed weekly. After isoflurane anesthesia, the rats were sacrificed. Blood samples, as well as testicular and epididymis tissue samples, were used for biochemical and microscopical evaluations.

### 2.2. Measurement of Serum Testosterone, FSH, and LH Concentrations

Serum testosterone, follicle-stimulating hormone (FSH), and luteinizing hormone (LH) concentrations were measured by using enzyme-linked immunosorbent assay (ELISA) kits. Rat testosterone (Catalog no: EA0023Ra, ELISA kit Bioassay Technology Laboratory), Rat FSH (Catalog no: EA0015Ra, Bioassay Technology Laboratory, Shanghai, China), and Rat LH ELISA (Catalog no: EA0013Ra, Bioassay Technology Laboratory) kits were used for hormone levels analysis according to the kit procedure. Testosterone results were given as nmol/L and FSH and LH were given as mIU/L.

### 2.3. Measurement of Testicular MDA, GSH, and SOD Levels

MDA levels were determined using a commercial kit (E-BC-K025-M, Elabscience, Houston, TX, USA). MDA, one of the degradation products of lipid peroxidation, reacts with Thiobarbituric acid (TBA) to form a pink complex with an absorption maximum of 532 nm. The MDA levels in the tissue were calculated in nmol/g. GSH analysis in testicular tissue was performed according to the Beutler method [28]. The principle of the method is based on the fact that GSH in the analysis tube reacts with 5,5′-dithiobis-2-nitrobenzoic acid (DTNB) to give a yellowish color. The light intensity of this color was read in the spectrophotometer at a wavelength of 410 nm. The tissue homogenates were centrifuged, and a 10% TCA solution was added to the obtained supernatant, mixed, and centrifuged again to precipitate the proteins. The brightly colored supernatants were used for GSH analysis. The intensity of the color formed in the samples kept at room temperature for 5 min was read at 410 nm in the spectrophotometer, and the GSH levels in µmol/g in the tissue were determined using the glutathione standard curve. SOD activity was determined using the Sigma SOD Determination Kit (E-BC-K019-M, Elabscience, Houston, TX, USA). Absorbance values were read at 450 nm after incubating SOD activity with an enzyme-working solution. The SOD levels in the tissue were calculated in IU/g.

### 2.4. Sperm Count, Motility, and Morphology

Epididymal tissue samples from rats were placed in an Earle’s Balanced Salts solution with a Hepes buffer solution and processed for analysis of sperm count, motility, and morphology based on a previous study [27]. Briefly, epididymis tissue samples of rats were transferred into an Earle’s Balanced Salts solution with Hepes buffer solution added to them following dissection. A routine density gradient method was used to evaluate the spermatozoa. The supernatant was removed, and the pellet was diluted with a spermatozoa washing medium (SAGE, Newcastle upon Tyne, UK) and centrifuged. Then the pellet was diluted with a sperm preparation medium (SAGE, UK). The 10 μm pellet was used to count spermatozoa in a Macler counting chamber (Sefi cut out Medical Instruments, Haifa, Israel) with a photomicroscope to count them. For morphological evaluation, smears were prepared and then stained using the Diff-Quick kit (Medion Diagnostics, Grafelfing, Munich, Germany). In each slide, 100 spermatozoa were examined at 100× magnification with a photomicroscope (Zeiss A1 Axio Scope, Oberkochen, Germany) to evaluate the sperm morphology.

### 2.5. Tissue Processing for Light Microscopy

Testicular tissues were fixed with the Bouin solution for routine histological examinations. For immunohistochemical examinations, testicular tissues were fixed with a 4% paraformaldehyde (PFA) solution. Following fixation, the tissues underwent routine a paraffin embedding procedure [29]. Haematoxylin and eosin (H&E) stain was applied to the sections for routine histological evaluation. Periodic acid-Schiff (PAS) reaction was applied to reveal the structure of the basal membranes of seminiferous tubules. Seminiferous tubules in H&E-stained testicular sections were scored on the basis of modified Johnsen’s histopathological scoring parameters [30,31]. In addition, the epithelial thicknesses of 100 seminiferous tubules were measured in all sections using Image J (Image J software, National Institutes of Health) program.

### 2.6. Terminal Deoxynucleotidyl Transferase dUTP Nick End Labelling (TUNEL) Immunochemistry

The terminal deoxynucleotidyl transferase dUTP nick end labeling (TUNEL) method was applied to testicular tissue sections according to the kit‘s instruction manual given by the manufacturer (ApopTag Plus, In Situ Apoptosis Detection Kit, S7101, Merck Millipore, Darmstadt, Germany). In brief, after deparaffinization and rehydration, the sections were washed in PBS and then heated in a microwave oven in citrate buffer. After cooling, proteinase K (20 μg/mL) was dripped onto the tissue sections. The sections were washed in distilled water and soaked in a 3% hydrogen peroxide-prepared solution. After washing in PBS, a 10 μL stabilizing tampon was placed on the sections for 30 min at room temperature. Then, 10 μL Tdt enzyme was dripped, and sections were incubated for 1 h at 37 °C. The stop-wash buffer was dripped for 10 min at room temperature. After washing with PBS, 13 μL of anti-digoxigenin was dripped onto the sections. After incubating at room temperature for 30 min, sections were washed 4 times for 2 min in PBS. Then, 13 μL 3,3’-Diaminobenzidine (DAB) solution was dripped. Then, the sections were shaken with distilled water. For contrast staining, the sections were soaked in Mayer hematoxylene solution (J.T Baker, Center Valley, PA, USA). After washing in distilled water sections were mounted with entallan. Sections were imaged with a light microscope (Zeiss A1 Axio Scope, Oberkochen, Germany). The apoptotic index was estimated by dividing the total number of testicular tubules by the number of seminiferous tubules with 3 or more TUNEL-positive cells [32].

### 2.7. Proliferating Cell Nuclear Antigen (PCNA) Immunohistochemistry

Sections were subjected to PCNA immunohistochemistry to determine the number of proliferative cells and the proliferation index. After deparaffinization and rehydration, the sections were processed for PCNA immunohistochemistry as described in a previous study [32]. In brief, the sections were stored in a 3% hydrogen peroxide solution (Sigma-Aldrich, St. Louis, MO, USA) for 20 min. The sections were heated in a microwave oven at 200 W in ethylenediaminetetraacetic acid (EDTA) buffer (Sigma-Aldrich, USA) for 20 min. After washing with PBS 3 times, the sections were soaked in a 5% goat serum-blocking solution (Invitrogen, Waltham, MA, USA) for 10 min. Then, the primary rabbit anti-PCNA antibody (Catalog no: SY12-07, Novus Biologicals, Littleton, CO, USA) (1:50) was applied. Sections were kept in a humidified chamber at 4 °C overnight and washed 3 times with PBS for 5 min each time. Biotin-labelled goat anti-rabbit secondary antibody (Catalog no: A16100, Invitrogen, Waltham, MA, USA) (1:500) was dropped onto the sections and kept at 37 °C for 30 min. Streptavidin peroxidase (Invitrogen, Waltham, MA, USA) was instilled into the sections and left for 10 min. After washing 3 times with PBS, the sections were soaked in 3,3′-diaminobenzidine (DAB) chromogen (1 mL DAB substrate + 30 μL DAB chromogen) for 5 min. After washing with distilled water, the sections were stained with Mayer’s hematoxylin solution for contrast staining. The sections were then mounted with Kaiser’s glycerol gelatin (Catalog no: 1.09242, Sigma-Aldrich, Darmstadt, Germany). The sections were examined under a light microscope (A1 Axio Scope, Oberkochen, Zeiss, Germany). To determine the proliferation index, the number of PCNA-positive cells in 20 tubules in each section was divided by the total number of cells [32].

### 2.8. Immunohistochemistry of ZO-1 and Occludin

The 4% PFA-fixed tissue sections were deparaffinized and rehydrated with alcohol solutions. Sections were then soaked in a 3% hydrogen peroxide solution (Sigma-Aldrich, St. Louis, MO, USA) for 20 min to block endogenous enzymes. After washing with PBS, the sections were heated in a microwave at 200 W in an EDTA buffer (Sigma-Aldrich, St. Louis, MO, USA). The sections were cooled for 30 min at room temperature and stored 3 times for 5 min each in PBS followed by 10 min in 10% buffered goat serum (Invitrogen, USA). The sections were treated with rabbit anti-ZO-1 (Catalog no: 61-7300, Invitrogen, Waltham, MA, USA) and rabbit antioccludin (1:100) (Catalog no: 71-1500, Invitrogen, Waltham, MA, USA) antibodies (overnight, at +4 °C). After washing 3 times in PBS for 5 min, a biotin-labelled antirabbit secondary antibody (Catalog no: 65-6140, Invitrogen, Waltham, MA, USA) (1:1000) was dropped onto the sections. After soaking in PBS 3 times and 5 min, streptavidin-peroxidase (Invitrogen, Waltham, MA, USA) was applied to the sections. After washing in PBS, AEC Single/Plus Chromogen (Abcam, Cambridge, UK) was dropped on, and sections were shaken with distilled water and mounted with Kaiser’s glycerol gelatin (Catalog no: 1.09242, Sigma-Aldrich, Darmstadt, Germany). Sections were imaged using a light microscope (A1 Axio Scope, Oberkochen, Zeiss, Germany). The intensity of immunoreactivity of both ZO-1 and occludin was calculated using the Image J program (1.44 software, National Institutes of Health).

### 2.9. ZO-1, Occludin and NOX-2 Immunofluorescence Analysis

The sections were kept in 3% hydrogen peroxide and washed with PBS. Then, sections were incubated with rabbit anti-ZO-1(1:100) ZO-1(Catalog no: 61-7300, Invitrogen, Waltham, MA, USA), rabbit antioccludin (1:100) (Catalog no: 71-1500, Invitrogen, USA), and rabbit anti-NOX-2 primary antibodies (1:100) (Catalog no: NBP2-41291, Novus, Bio-Techne, Minnesota, USA). Slides were washed with PBS and then incubated with AF 488 (Catalog no: ab150077, Abcam, USA) labeled goat antirabbit secondary antibody (1:1000). After the sections were washed with PBS for the last time, they were mounted with a 4′6-diamino-2-phenylidol (DAPI) solution (Catalog No: ab104139, Abcam, Boston, MA, USA). The sections were imaged with a fluorescence microscope (Zeiss Axio Scope.A1 microscope with fluorescence attachment Zeiss AxioCam MRc 5 camera). The fluorescence intensity in the photographed sections was calculated using Image J (Image J software, National Institutes of Health) program.

### 2.10. Tissue Processing for Transmission Electron Microscopy

Testis tissue samples were fixed with buffered 2.5% glutaraldehyde solution (in 0.1 M PBS, pH 7.2) and processed for routine transmission electron microscopical preparation according to the previously published protocol [32]. Sections were analyzed under transmission electron microscope (TALOS L 120 C, Thermo Scientific Fisher, Eindhoven, The Netherlands). 

### 2.11. Statistical Analysis

Data were analysed with one-way ANOVA and Tukey’s multiple comparison tests with *p* < 0.05 considered significant. Statistical analysis was performed using Graph Pad Prism 8.0 (San Diego, CA, USA).

## 3. Results

### 3.1. Serum Testosterone, FSH, and LH Levels

**Figure 1:**
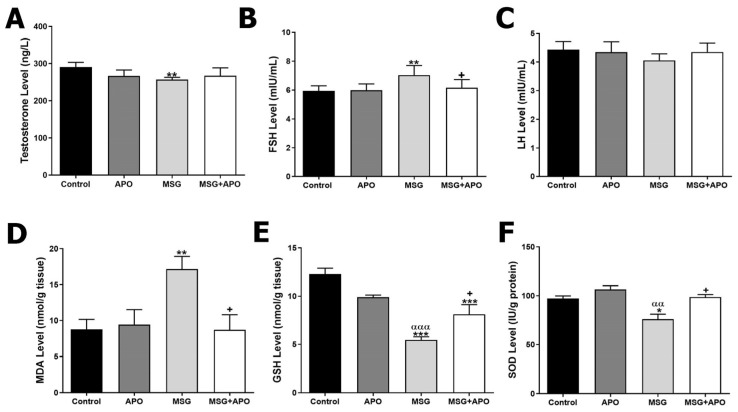
Testosterone (**A**), FSH (**B**), LH (**C**), MDA (**D**), GSH (**E**) levels, and SOD (**F**) activity in the experimental groups. * *p* < 0.05, ** *p* < 0.01, *** *p* < 0.001 vs. control group; ^+^ *p* < 0.05 vs. MSG group; ^αα^ *p* < 0.01, ^ααα^ *p* < 0.001 vs. APO group.

Testosterone levels in the MSG group were lower compared to the control group. Although the MSG + APO group showed an increase in testosterone levels compared to the MSG group, this increase was not statistically significant (Figure 1A). The FSH level was higher in the MSG group compared to the control group. The FSH level in the MSG + APO group was decreased compared to the MSG group (Figure 1B). Decreased levels of LH in the MSG group and increased levels of LH in the MSG + APO group were not statistically significant compared to the experimental groups (Figure 1C).

### 3.2. Testicular MDA, GSH, and SOD Levels

The tissue MDA level was increased in the MSG group compared to the control group. On the other hand, when comparing the MSG + APO group with the MSG group, a significant decrease in MDA was observed (Figure 1D). GSH and SOD levels were decreased significantly in the MSG group when compared with the control and APO groups, respectively. A significant increase in GSH and SOD levels was revealed in the MSG + APO group compared with the MSG group (Figure 1E,F).

### 3.3. Sperm Count, Motility, and Morphology

**Figure 2:**
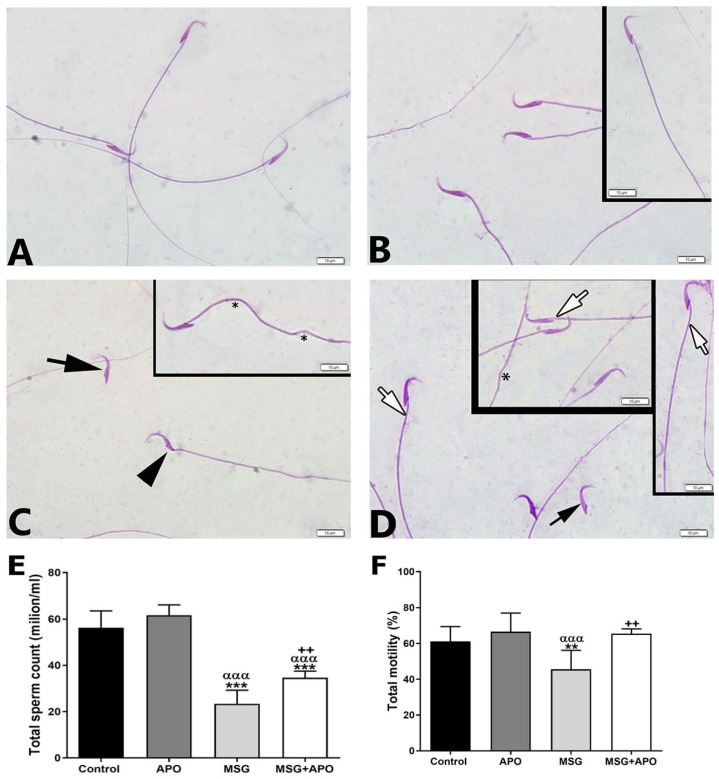
Representative light micrographs (**A**–**D**) of sperm morphology and graphs of total sperm count (**E**) and motility (**F**) in the experimental groups. Normal sperm morphology in the control (**A**) and APO groups (**B**). Spermatozoa with disturbed morphology with tailless sperm head (black arrow) and neck (black arrowhead) in MSG group (**C**). Inset: tail abnormality (*). Spermatozoa with normal morphology (white arrow) and disturbed morphology with head structure of tailless spermatozoa (black arrow) were observed in MSG + APO group (**D**). Inset: spermatozoa with normal morphology (white arrow) and tail abnormality (*). Scale bar: 50 μm. Diff-Quik staining. Total sperm count (**E**) and motility (**F**) graphs in the experimental groups. ** *p* < 0.01, *** *p* < 0.001 vs. control group; ^++^ *p* < 0.01 vs. MSG group; ^ααα^ *p* < 0.001 vs. APO group.

Normal morphology was observed in sperm samples in the control group and APO groups (Figure 2A,B). A large number of spermatozoa with morphological abnormalities were observed in the MSG group (Figure 2C). In the MSG + APO group, few abnormal sperm were detected, as a majority of sperms were of normal morphology (Figure 2D). 

The sperm count and motility were lower in the MSG group compared to the control and APO groups. Those parameters were increased significantly in the MSG + APO group compared to the MSG group (Figure 2E,F).

### 3.4. Testicular Weight/Body Weight Ratio

**Figure 3:**
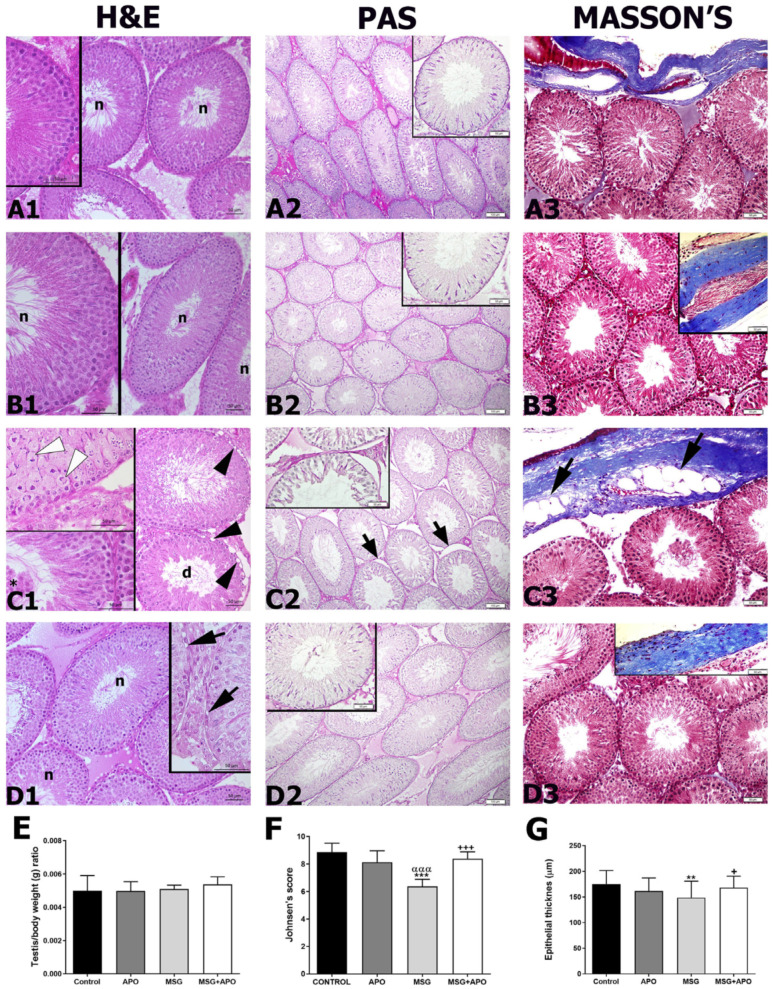
Representative light micrographs of testis tissue (**A1**–**D3**), testis/body weight (**E**), histopathological Johnsen’s score (**F**) and thickness of seminiferous tubules (**G**) in the experimental groups. The regular morphology of the germinal epithelium is observed in the control (**A1**) and APO (**B1**) groups. Vacuolization (black arrowhead) in basement membranes and few spermatozoa were observed in the lumen of degenerative seminiferous tubules in the MSG (**C1**) group. Inset: cell division (white arrowhead) and immature germ cells (asterisk) were observed in the seminiferous tubule epithelium. Improved morphology of seminiferous tubules was observed in the MSG + APO (**D1**) group. Inset: vacuolization (black arrow) was observed in a small number of tubules. d: degenerative tubule, n: normal tubule; H & E staining. Regular PAS-positive stained basement membrane morphology was observed in the control (**A2**), APO (**B2**), and MSG + APO (**D2**) groups, but irregular PAS-positive stained basement membrane morphology (arrow) was observed in the MSG group (**C2**). PAS reaction. The morphology of the testicular tubules and tunica albuginea was normal in the control (**A3**), APO (**B3**), and MSG + APO (**D3**) groups. However, in the MSG group, fat tissue (arrow) was observed in the tunica albuginea, and normal morphology of the tubular stroma was also observed. Masson’s trichrome. Scale bar: 50 μm. Testis/body weight ratio (**E**), histopathological Johnsen’s score (**F**), and thickness of seminiferous tubules (**G**) in the experimental groups. ** *p* < 0.01, *** *p* < 0.001 vs. control group; ^+^ *p* < 0.05, ^+++^ *p* < 0.001 vs. MSG group; ^ααα^ *p* < 0.001 vs. APO group.

Although the testicular weight/body weight ratio of the MSG group was observed to be lower compared to the other experimental groups, this decrease was not statistically significant. There was no statistical difference in the testicular weight/body weight ratio between the experimental groups (Figure 3E).

### 3.5. Histopathological Results

Light microscopical examination of testicular tissue by H&E staining revealed normal morphology of the seminiferous tubules in the control group. (Figure 3(A1)). Intact testicular morphology was observed in the APO group (Figure 3(B1)). In the MSG group, the germinal epithelia of the seminiferous tubules were disorganized. Vacuoles indicating prominent tissue damage were detected in the basal compartment of the seminiferous tubules (Figure 3(C1)). Those morphological disturbances were reflected in a significantly high histopathological score for this group. In the MSG + APO group, the majority of seminiferous tubules reflected the normal morphology, with a limited number of degenerated seminiferous tubules (Figure 3(D1)). Compared with the MSG group, the histopathological score was significantly low in the control and APO groups. The histopathological score significantly increased in the MSG group and was significantly decreased in the MSG + APO group, compared to the MSG group (Figure 3F). In addition, the thickness of the seminiferous tubules in the MSG group was low compared to the other experimental groups. In the MSG + APO group, there was a significant increase in tubule thickness compared to the MSG group (Figure 3G).

The control and APO groups presented a strong PAS-positivity (Figure 3(A2,B2)) for the basement membranes of seminiferous tubules with regular morphology. Deteriorated basement membranes of seminiferous tubules of MSG resulted in a decreased PAS-positivity of sections. (Figure 3(C2)). PAS-positivity of the MSG + APO group was nearly equivalent to the control group, with a small number of disturbed seminiferous tubules presenting detached basement membranes (Figure 3(D2)). The morphologies of testicular tubules and tunica albuginea were normal in the control group (Figure 3(A3)), APO group (Figure 3(B3)), and MSG + APO group (Figure 3(D3)). However, in the MSG group (Figure 3(C3)), fat tissue was observed in the tunica albuginea, and the morphology of the tubular stroma was also normal.

### 3.6. Results for PCNA

**Figure 4:**
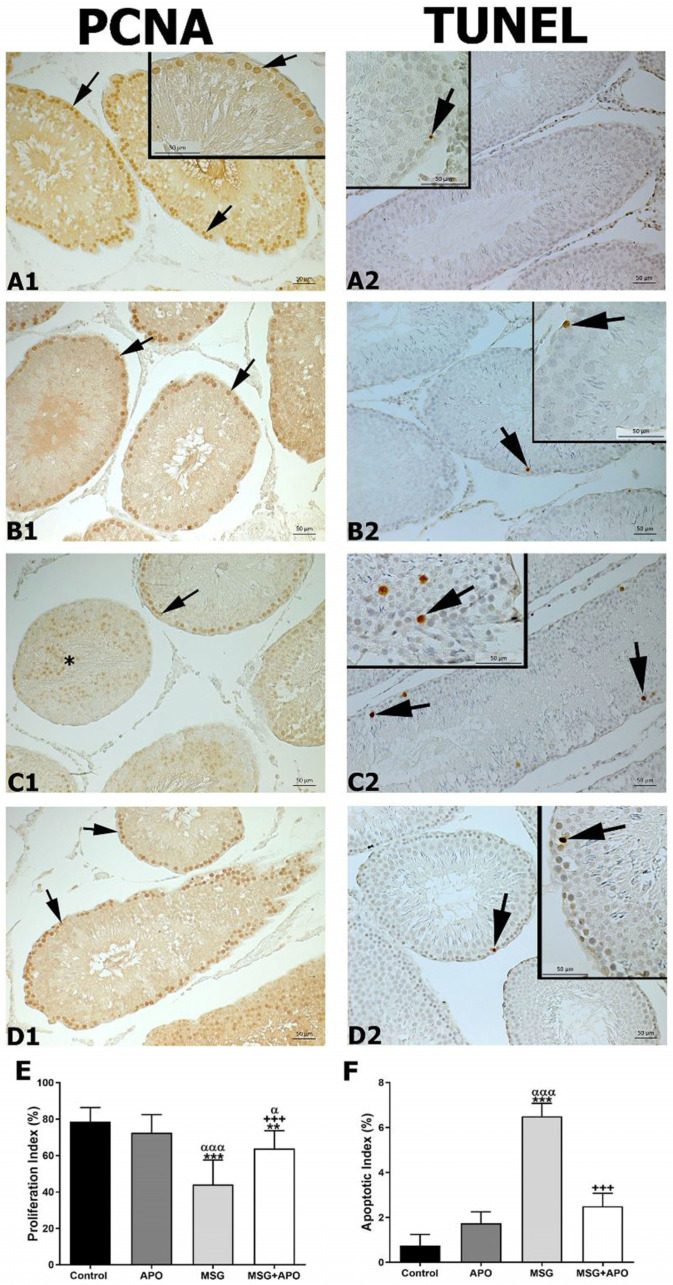
Representative photomicrographs of PCNA immunostained (**A1**–**D1**) and TUNEL stained (**A2**–**D2**) testis tissues, proliferative (**E**), and apoptotic (**F**) indexes in the experimental groups. Numerous PCNA-positive (arrow) spermatogenic cells are seen in seminiferous tubules of the control (**A1**) and APO (**B1**) groups. Decreased PCNA-positive cells (arrow) in the seminiferous tubules and PCNA-positive spermatogenic cells (asterisk) in the lumen of seminiferous tubules are seen in the MSG group (**C1**). Numerous PCNA-positive spermatogenic cells (arrow) are seen in the seminiferous tubules of the MSG + APO group (**D1**). A few TUNEL-positive spermatogenic cells (arrow) are seen in the control (**A2**) and APO (**B2**) groups. An increased number of TUNEL-positive cells (arrow) in the seminiferous tubules were observed in the MSG group (**C2**). A decreased number of TUNEL-positive cells (arrow) was observed in the seminiferous tubules of the MSG + APO group (**D2**). Scale bar: 50 μm. Proliferative (**E**) and apoptotic (**F**) indexes of the experimental groups. ** *p* < 0.01, *** *p* < 0.001 vs. control group; ^+++^ *p* < 0.001 vs. MSG group; ^α^ *p* < 0.05, ^ααα^ *p* < 0.001 vs. APO group.

A large number of PCNA-positive cells in the seminiferous epithelium, as dark brown, were observed in the control and APO groups (Figure 4(A1,B1)). In the MSG group, a decrease in the number of PCNA-positive cells of the seminiferous epithelium was noticed (Figure 4(C1)). Also, PCNA-positive spermatogenic cells in the lumen of seminiferous tubules are seen in the MSG group. The proliferation index, lowest in the MSG group, was increased in the MSG + APO group compared to the MSG group (Figure 4E). The MSG + APO group also showed an increase in PCNA-positive cells in the seminiferous epithelium (Figure 4(D1)).

### 3.7. Results for TUNEL Immunocytochemistry

In the control and APO groups, TUNEL-positive cells were low (Figure 4(A2,B2)). The number of TUNEL-positive cells was higher in the MSG group than in the other experimental groups (Figure 4(C2)). TUNEL-positive cells were fewer in the MSG + APO group than in the MSG group (Figure 4(D2)). The apoptotic index was higher in the MSG group than in the other experimental groups. There was a comparative decrease in this index in the MSG + APO group (Figure 4F).

### 3.8. Results for NOX-2 Immunofluorescence

**Figure 5:**
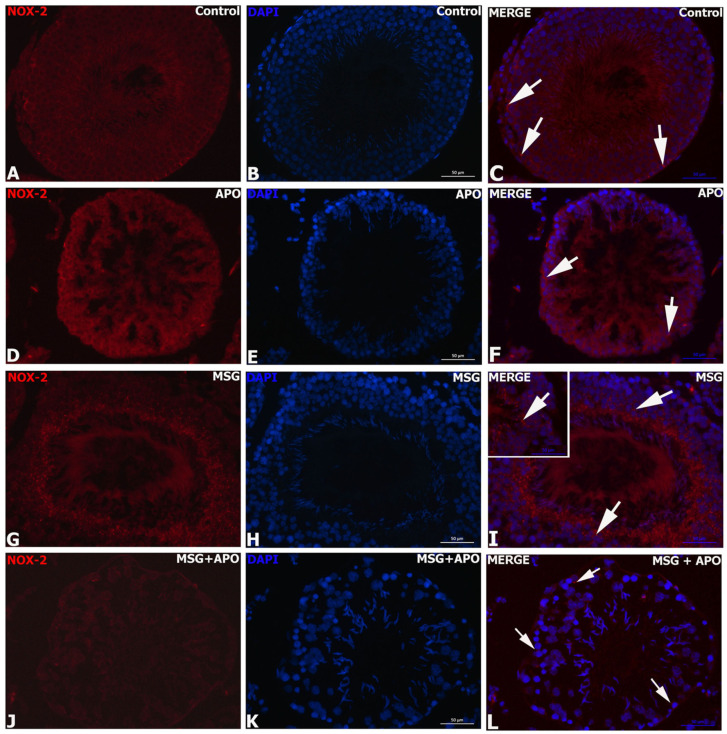
Immunofluorescence analysis of NOX-2 in experimental groups (**A**–**L**). The nuclei were labeled with DAPI (blue). NOX-2 expression was similar in the control, APO, and MSG + APO groups. An increased number of NOX-2-positive cells (red) were observed in the MSG group. White arrows indicate NOX-2-positive cells. Scale bar: 50 μm.

Weak NOX-2 immunopositivity was observed in the control (Figure 5A–C) and APO groups (Figure 5D–F). The highest immunoreactivity was observed in the MSG group (Figure 5G–I), whereas a decrease was detected in the MSG + APO group (Figure 5J–L). Negative controls of the NOX-2 immunofluorescence analysis were shown in Figure S1

### 3.9. Results for ZO-1 and Occludin Immunohistochemistry

**Figure 6:**
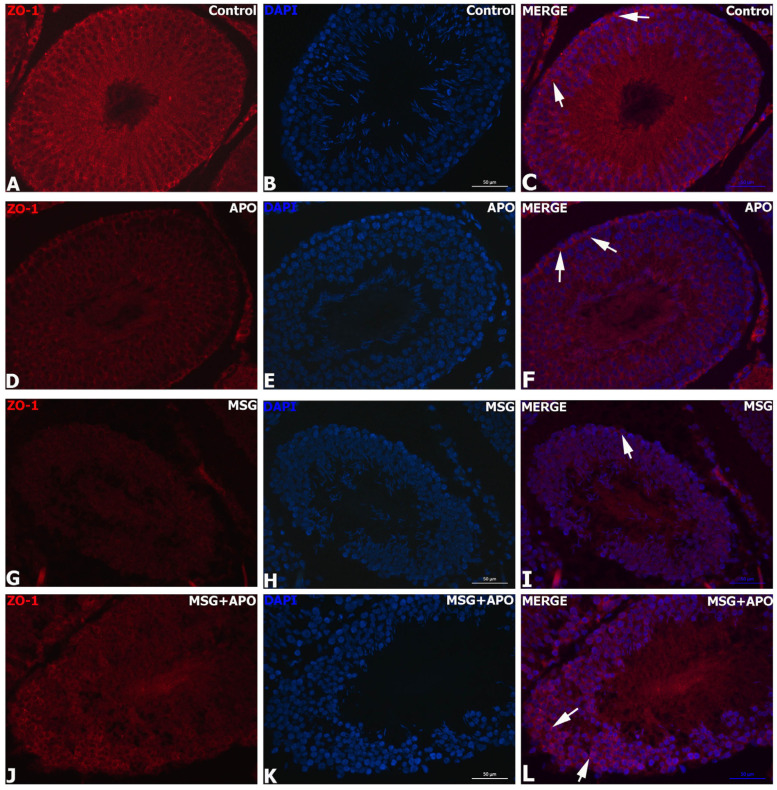
Immunofluorescence analysis of ZO-1 in experimental groups (**A**–**L**). Representative photomicrographs showing immunodetection of ZO-1 (red) in the experimental groups. Cell nuclei were labeled with DAPI (blue). ZO-1-positive (white arrow) basolateral and apical regions of Sertoli cells were observed in each experimental group. ZO-1-positive cells were decreased in the MSG group. Scale bar: 50 μm.

**Figure 7:**
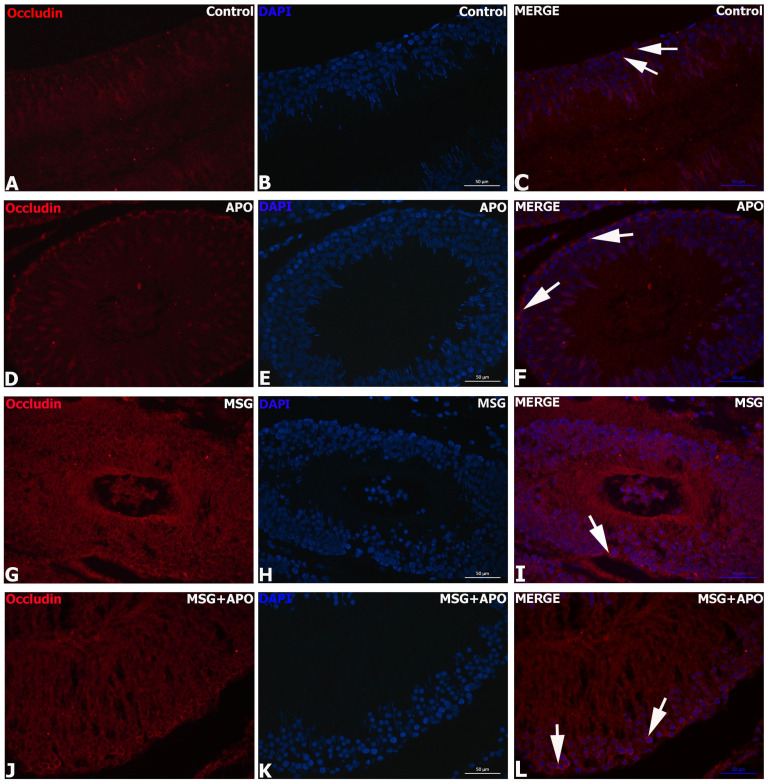
Immunofluorescence analysis of occludin in experimental groups (**A**–**L**). Representative photomicrographs showing immunodetection of occludin (red) in the experimental groups. Cell nuclei were labeled with DAPI (blue). Occludin-positive (white arrow) basolateral and apical regions of Sertoli cells were observed in each experimental group. Occludin-positive cells were decreased in the MSG group. Scale bar: 50 μm.

**Figure 8:**
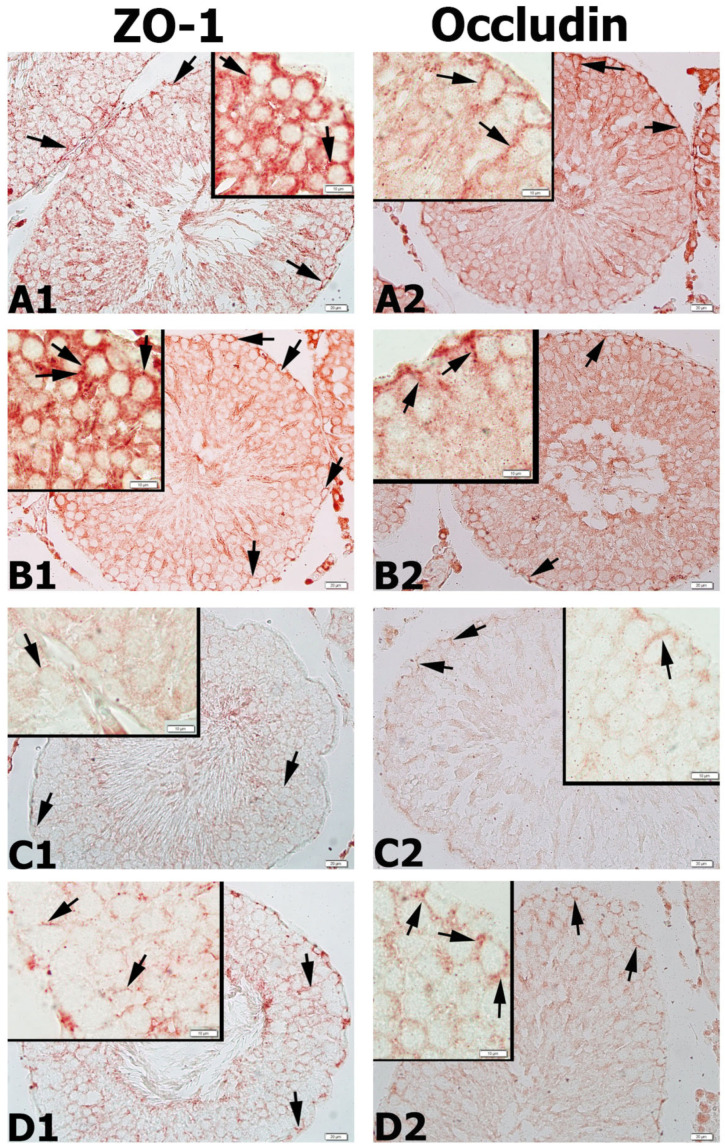
Representative photomicrographs of ZO-1 (**A1**–**D1**) and occludin (**A2**–**D2**) immunostained testis tissue samples. Similar ZO-1 and occludin immunostaining in the basolateral regions of Sertoli cells was observed in the control (**A1**,**B1**) and APO (**A2**,**B2**) groups. Decreased ZO-1 (**C1**) and occludin (**C2**) immunostaining were observed in the basolateral regions of Sertoli cells in the MSG group. Increased ZO-1 (**D1**) and occludin (**D2**) immunostaining in the basolateral regions of Sertoli cells were observed in the MSG + APO group. Black arrows show ZO-1 and occludin-positive regions of the cells. Scale bar: 50 μm.

**Figure 9:**
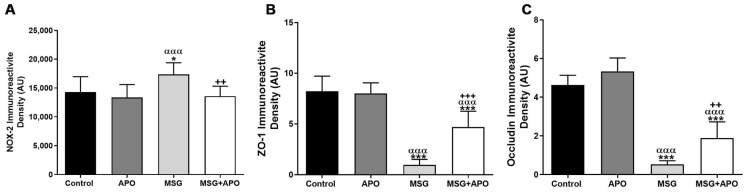
NOX-2 (**A**), ZO-1 (**B**), and occludin (**C**) immunoreactivity densities in the experimental groups. * *p* < 0.05, *** *p* < 0.001 vs. control group. ^++^ *p* <0.01; ^+++^ *p* <0.001 vs. MSG group; ^ααα^ *p* < 0.001 vs. APO group. Values are given as the mean ± SD.

ZO-1 and occludin positivities were detected as a dark red color in the basolateral cytoplasm of Sertoli cells located in the seminiferous tubule of the germinal epithelium. While the intensities of ZO-1 and occludin immunoreactivity were highest in the control group (Figure 6A–C, Figure 7A–C, Figure 8(A1,A2) and Figure 9A,B) and APO group (Figure 6D–F, Figure 7D–F and Figure 8(B1,B2)) and a decrease in the distribution of immunoreactivities was observed in the MSG group (Figure 6G–I, Figure 7G–I and Figure 8(C1,C2)). Increases in ZO-1 and occludin positivities were observed in the MSG+APO group, compared to the MSG group (Figure 6J–L, Figure 7J–L and Figure 8(D1,D2)).

### 3.10. Transmission Electron Microscopical Results

**Figure 10:**
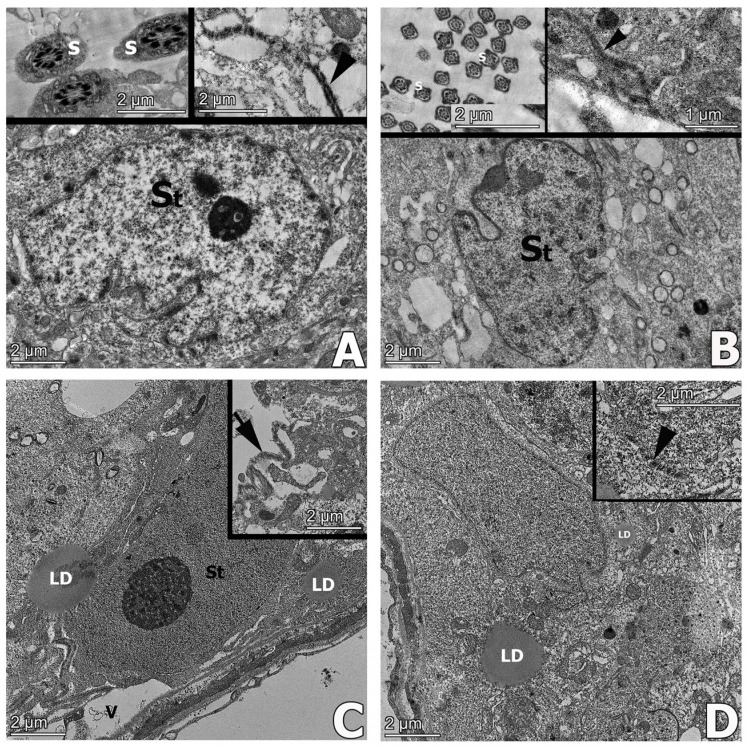
Representative electron micrographs of testis samples in the experimental groups. The regular morphology of tight junctions (arrow) between adjacent Sertoli cells and numerous spermatozoa (s) were observed in the control (**A**) and APO (**B**) groups. Degenerated tight junctions (arrowhead) and lipid droplets (LD) in the cytoplasm were observed in the MSG group (**C**). Normal organization of seminiferous tubules and tight junction morphology (arrowhead) with few lipid droplets (LD) in the cytoplasm were observed in MSG + APO (**D**) group. St: Sertoli cell, s: spermatozoa, LD: lipid droplets. Ultrathin sections were contrasted with uranyl-acetate-lead citrate.

In the control and APO groups, normal ultrastructure of seminiferous tubular germinal epithelium presenting numerous spermatozoa, and Sertoli cells interconnected with tight junctions were observed (Figure 10A). The APO group had a similar ultrastructure as with the control group (Figure 10B). In the MSG group, vacuolization and lipid droplets in the germinal epithelial cells of the seminiferous tubule, separations between the Sertoli–Sertoli cell junctions and deteriorated basal lamina of the seminiferous tubules were observed (Figure 10C). The ultrastructure of the MSG + APO group reflected the normal organization of seminiferous tubules with few lipid droplets in the cytoplasm (Figure 10D).

## 4. Discussion

In this study, the healing effect of APO on MSG-induced testicular damage has been evaluated by biochemical, microscopical, and immunohistochemical parameters. Testosterone and LH levels decreased and FSH levels increased, oxidative stress parameters such as MDA level increased, while GSH and SOD levels decreased in the MSG group. In MSG-induced testicular injury, the histopathological score and the number of damaged seminiferous tubules and spermatozoa were increased. The proliferative index was low and the apoptotic index was higher in the MSG group. The germinal epithelium of the seminiferous tubules was decreased in thickness. Those degenerative changes have been reversed by the administration of APO. The MSG group depicted a decrease in the distribution of ZO-1 and occluding as tight junction proteins of the blood-testis barrier. MSG affected the oxidative stress markers. In this group, there was an increase in the distribution of the protein NOX-2, which was involved in the synthesis of ROS. Ultrastructure examinations of the MSG group indicated prominent damage in the cytoplasm of seminiferous tubule germinal epithelial cells of the seminiferous tubules with separated tight junctions. Biochemical, histological, immunohistochemical, and ultrastructural analyses showed that these parameters improved with APO treatment in the MSG + APO group.

Experimental studies have shown that oral ingestion of MSG can be toxic to reproduction; however, due to the high doses used in the studies, the toxic effects on the reproductive system due to consumption of MSG were unlikely to translate to human consumption [33,34] The human ingested daily dose of MSG is thought to be between 1200 and 3000 mg/kg [35]. In this study, the dose of 120 mg/kg body weight of MSG was used for experimental design, based on an allometric extrapolation of the average daily intake of this substance in humans [5].

The hypothalamic–pituitary–gonadal (HPG) axis plays a key role in processes related to the development and maturation of the male reproductive system. FSH is a factor in the maintenance of intragonadal testosterone synthesis and spermatogenesis. LH plays a role in the development of the male genital system and the process of sex determination by stimulating Leydig cells [36]. The stimulation causes Leydig cells to secrete the necessary levels of testosterone. The proper testosterone level is controlled by GnRH activity through a negative feedback mechanism [36]. With this mechanism, the HPG axis functions in a controlled manner [37,38]. In some population-based studies, sex hormones have been shown to affect sperm morphology, concentration, and motility. In a study of subfertile couples, an inverse relationship was found between LH and sperm motility and morphology [39]. In clinical studies, FSH levels were found to correlate negatively with sperm concentration [40]. In other experimental studies, following the administration of MSG, a decrease in sperm motility, concentration, and LH hormone levels was revealed. Studies have also shown that MSG causes a decrease in testosterone hormone levels and sperm concentrations [34,41]. In our present study, FSH, LH, and testosterone levels in the APO group were similar to the control group. A statistically significant decrease in testosterone hormone was observed in the MSG group. A significant increase in FSH hormone levels was observed in the MSG group. In the MSG + APO group, the FSH level was lower than in the group MSG. Although a decrease in LH hormone was observed in the MSG group, there was no statistical difference in LH hormone between the experimental groups. MSG could do damage to Leydig cells for testosterone secretion. Decreased testosterone and LH levels are also associated with sperm count. Thus, low testosterone and LH levels could be related to decreased sperm count and abnormal sperm morphology [42]. In contrast, an elevated FSH level indicates that there is a disturbance in spermatogenesis causing altered FSH signaling. MSG has been shown that it may cause a disturbance in hormonal pathways in the hypothalamus [4]. As a result of our study, it was observed that administration of MSG interfered with the HPG axis by affecting FSH, LH, and testosterone levels, as well as testicular and sperm morphology, sperm concentration, and motility. Administration of apocynin reduced the impaired testosterone, FSH, and LH levels to near normal levels and this improvement in hormone levels also affected the morphological parameters.

SOD is an important enzyme that protects cells from damage caused by internal and external superoxide ions [43]. MDA is an aldehyde formed by free radicals during lipid peroxidation and is an indicator of both cell membrane damage and the severity of the effect of oxygen radicals on reactive cells. A decrease in SOD and an increase in MDA can trigger oxidative stress, leading to cell damage and cell death [24]. GSH is involved in the reduction of ROS such as hydrogen peroxide in the cell [44]. NOX is a source of ROS and apocynin is known as a NOX inhibitor [45]. In studies of MSG-induced testicular injury, MDA levels in testicular tissue increased and SOD levels decreased when MSG was administered [4,46]. In another study, ingestion of MSG was also shown to decrease GSH levels in testicular tissue [4]. In a study in which apocynin was used as a healing agent, it was shown that apocynin reduced MDA levels, which were increased due to tissue damage and caused an increase in GSH and SOD levels in the testicular damage group [27]. In another experimental study of the hypoxia-induced testicular degeneration model, NOX-2 activation and MDA levels were increased and GSH levels were decreased due to the effect of hypoxia. However, testicular tissue damage was shown to be reduced by the application of APO [47]. Our present study concluded an increase in MDA levels with a decrease in GSH and SOD levels in the MSG group. We could suggest that APO improved these parameters in the MSG + APO group through NOX-2 inhibition.

Some preclinical studies have revealed the toxic effects of MSG on many organ systems, such as the liver, kidneys, and reproductive system [3]. An experimental study states that MSG can cause infertility due to its harmful effect on the ovaries and oocytes [48]. The toxic effect of MSG on the testes with an imbalance of sex hormones has been noted [34]. In a similar study, the levels of oxidative stress markers were increased in the testicular tissues of rats administered MSG, and testicular morphology and sperm morphology were also negatively affected by MSG [41]. It has been observed that MSG leads to a degeneration in testicular morphology with a negative effect on testosterone [4,49]. In testicular injury models, a decrease in the thickness of the seminiferous tubule germinal epithelium was demonstrated [50,51]. In an MSG-induced testicular injury model the thickness of the seminiferous tubule epithelium was decreased [52]. In our study, the thickness of the seminiferous tubular germinal epithelium of the MSG group was decreased compared to the other experimental groups. There was an increase in the germinal epithelial thickness of the MSG + APO group compared to the MSG group.

MSG is stated to lead to obesity, as an appetizer [53]. There are question marks about whether MSG consumption can be a risk factor in the rapid increase in epidemic obesity. In an open-cohort study among Chinese adults, it was shown that those who used MSG had a higher body mass index compared with those who did not use MSG [54]. In other experimental studies, it has been observed that the weight of MSG-given rats increased and made them prone to obesity [55,56]. However, in our study, there was no statistically significant difference in body weight between the experimental groups, weighted every week during the experiment. In a related study where MSG has been administrated to rats, a decrease was observed in testicular weights in the MSG group compared to the control group [4,52]. However, testicular weight/body-weight ratios of our current study, indicated a decrease in the MSG group compared to other experimental groups, as statistically insignificant. 

In studies examining the effects of MSG on sperm concentrations, MSG was found to cause a decrease in sperm viability [4,49,52]. In another testicular damage model, the curative effect of apocynin on sperm parameters was examined, and an increase in sperm concentration was observed in the apocynin healing group compared to the damage group [27]. In our study, when comparing sperm counts between experimental groups, it was found that the sperm count was lowest in the MSG group, while the sperm count in the MSG + APO group was statistically higher than in the MSG group.

It is stated that the use of apocynin is safe in animal studies. In addition, APO has potent antioxidant and anti-inflammatory effects in many experimental models [57]. Experimental testicular injury models reported the effect of apocynin on the reduction of oxidative stress [58,59]. In chemotherapy-based experimental studies, the healing effect of apocynin was also highlighted on testes tissue. In these studies, it was observed that apocynin leads to a decrease in the number of apoptotic cells and an increase in testosterone levels [27,60]. As one of the factors of male infertility, oxidative stress leads to sperm dysfunction. Oxidative stress-induced spermatozoon damage affects 30–80% of infertile men [61]. Enzymes in the NOX family are responsible for cellular ROS synthesis, and apocynin is a NOX inhibitor [45]. In an experimental model of testicular injury in which apocynin was used as a healing agent, the highest NOX-2 immunoreactivity was observed in the testicular injury group. APO treatment concluded in a decrease in the number of NOX-2 immunopositive cells [27]. Literature data revealed a limited number of experimental testicular damage studies evaluating the amount and distribution of NOX-2. In our study, NOX-2 immunoreactivity was highest in the MSG group, with a decreased immunoreactivity in the MSG + APO group compared to the MSG group. There was no significant difference between the APO group and the control group, presenting the lowest NOX-2 immunoreactivity.

Apoptosis is the programmed cell death, which is essential for tissue homeostasis [62]. Regulation of apoptosis is a critical factor in the development, differentiation, and function of germ cells [61]. Exposure to factors that lead to oxidative stress negatively affects spermatogenesis by negatively affecting the structure of chromatin in spermatozoa [63]. Infertile men have been found to have higher oxidative stress and apoptotic cells in their seminal fluid compared to fertile men. In experimental models of testicular damage, the increased number of apoptotic cells in damaged testis tissue was reversed by the administration of APO as a curative substance [23,51]. In an experimental study of testicular injury, apocynin was shown to have a healing effect on DNA damage caused by NOX -induced oxidative stress [64]. Similarly, in our study, the proliferation index was decreased in the MSG group, with an increase in the apoptotic index. In the MSG + APO group, a decrease in the apoptotic index and an increase in the proliferative index were evaluated.

The blood-testis barrier protects the developing germ cells against the external environment and provides support for the germ cells to divide and develop, which is very important for fertility [65]. Sertoli cells, which are specialized epithelial cells found in the seminiferous tubules, form the basis of the blood-testis barrier with tight junctions. Occludin, ZO-1, ZO-2, and ZO-3 are molecules found in the tight junctions of the blood-testis barrier. ZO proteins are involved in binding the occludin proteins to the actin in the cytoskeleton [66,67]. It was observed that spermatogenesis was negatively affected, and atrophic seminiferous tubules formed in the occludin knockout mouse model. Our study revealed the highest immunoreactivities of ZO-1 and occludin in the control group and APO group, whereas the MSG group had significantly lower immunoreactivity. ZO-1 and occludin immunoreactivities were higher in the MSG + APO group, compared to the MSG group.

Transmission electron microscopic examinations of experimental studies depicted degenerated testicular germinal epithelial cells and lipid droplets [41,54] with deteriorated tight junctions in the blood-testis barrier [68]. In our study, transmission electron microscopic examinations revealed normal testicular seminiferous tubule ultrastructure in the control and APO groups. Vacuolization, lipid droplets, and degenerations in the tight junctions of the blood-testis barrier were evident in the MSG group. In the MSG + APO group, apocynin was found to have a healing effect at the level of ultrastructural damage.

## 5. Conclusions

Our study concluded with a regulating role of APO, for the formation of reactive oxygen derivatives and the HPG axis in rat testicular injury induced by MSG. APO maintained the balance between the proliferative and apoptotic indexes, preserved the integrity of the blood-testis barrier, and contributed to the healing of testicular tissue damage caused by oxidative damage. It is also thought that apocynin administration may have a healing effect on infertility by reversing the harmful effects of MSG both on sperm motility and number.

## Data Availability

The data used to support the findings of this study are available from the corresponding author upon request.

## Supplementary Materials

The following supporting information can be downloaded at: https://www.mdpi.com/article/10.3390/life13030822/s1. Figure S1: Negative control (without primary antibody) of NOX-2 immunofluorescence analysis.
