# Supplementary figures and images for: Apocynin Ameliorates Monosodium Glutamate Induced Testis Damage by Impaired Blood-Testis Barrier and Oxidative Stress Parameters
Source: Life (Basel). 2023 Mar 17;13(3):822. doi: 10.3390/life13030822 (PMC10052003; doi:10.3390/life13030822)

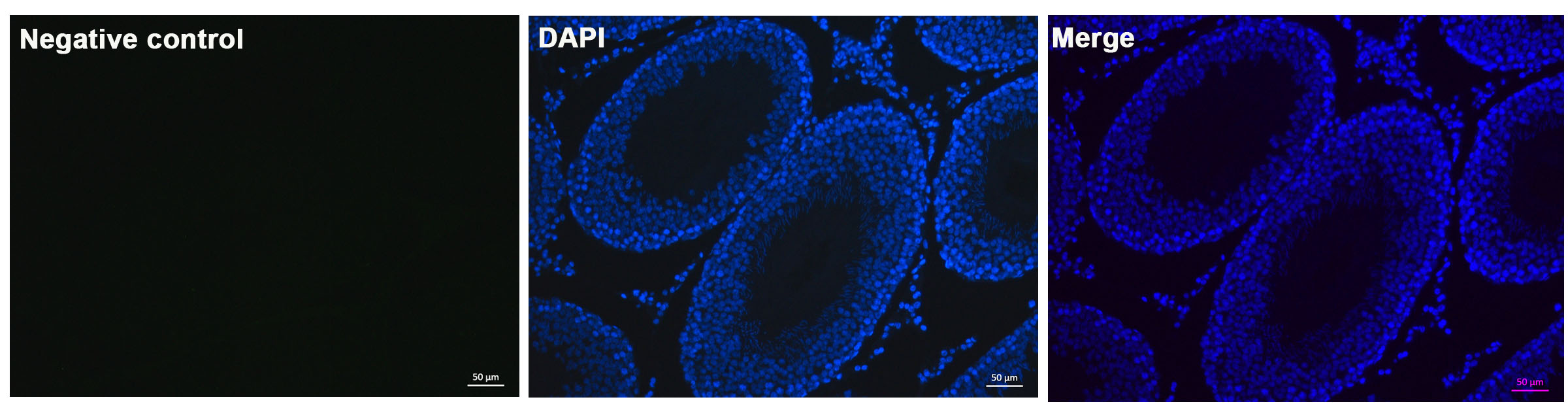

Supplement: Supplementary file 1 [file life-13-00822-s001.zip › Figure S1.jpg]
